# Material Characterization and Stress-State-Dependent Failure Criteria of AASHTO M180 Guardrail Steel: Experimental and Numerical Investigation

**DOI:** 10.3390/ma18112523

**Published:** 2025-05-27

**Authors:** Qusai A. Alomari, Tewodros Y. Yosef, Robert W. Bielenberg, Ronald K. Faller, Mehrdad Negahban, Zesheng Zhang, Wenlong Li, Brandt M. Humphrey

**Affiliations:** 1 Midwest Roadside Safety Facility, University of Nebraska-Lincoln, Lincoln, NE 68583, USA; tyosef2@unl.edu (T.Y.Y.); rbielenberg2@unl.edu (R.W.B.); rfaller1@unl.edu (R.K.F.); mnegahban@unl.edu (M.N.); 2 Department of Civil and Environmental Engineering, University of Nebraska-Lincoln, Lincoln, NE 68586, USA; 3 Mechanical and Material Engineering, University of Nebraska-Lincoln, Lincoln, NE 68586, USA; wli2@ujs.edu.cn; 4 Dimensional Control Systems, Troy, MI 48083, USA; zzszidane@gmail.com; 5 Burns and McDonnell Construction, Kansas City, MO 64114, USA

**Keywords:** guardrail steel, ductile fracture, stress-state-dependent failure, triaxiality, lode parameter, GISSMO, LS-DYNA

## Abstract

As a key roadside safety feature, longitudinal guardrail steel barriers are purposefully designed to contain and redirect errant vehicles to prevent roadway departure, dissipate impact energy through plastic deformation, and reduce the severity of vehicle crashes. Nevertheless, these systems should be carefully designed and assessed, as localized rupturing, especially near splice or impact locations, can lead to catastrophic failures, compromising vehicle containment, violating crash safety standards, and ultimately jeopardizing the safety of occupants and other road users. Before conducting full-scale crash testing, finite element analysis (FEA) tools are widely employed to evaluate the design efficiency, optimize system configurations, and preemptively identify potential failure modes prior to expensive physical crash testing. To accurately assess system behavior, calibrated material models and precise failure criteria must be utilized in these simulations. Despite the existence of numerous failure criteria and material models, the material characteristics of AASHTO M-180 guardrail steel have not been fully investigated. This paper significantly advances the FE modeling of ductile fracture in guardrail steel, addressing a critical need within the roadside safety community. This study formulates stress-state-dependent failure criteria and proposes advanced material modeling techniques. Extensive experimental testing was conducted on steel specimens having various triaxiality and Lode parameter values to reproduce a wide spectrum of complex, three-dimensional stress-state loading conditions. The test results were then used to identify material properties and construct a failure surface. Subsequent FEA, which incorporated the Generalized Incremental Stress-State-Dependent Damage Model (*GISSMO*) in conjunction with two LS-DYNA material models, illustrates the capability of the developed surface and material input parameters to predict material behavior under various stress states accurately. A parametric study was completed to further validate the proposed models, highlighting their robustness and reliability.

## 1. Introduction

Longitudinal barriers, such as W-beam steel guardrail systems, are crucial components in roadside safety systems that are widely used throughout the nation’s roads and highways to mitigate the severity of run-off-road crashes. Similar to other forms of safety hardware, these guardrail steel systems are essentially designed to contain and redirect errant vehicles while ensuring occupant safety. Specifically, these roadside safety features are purposefully designed to undergo large plastic deformations to properly dissipate the vehicle’s impact energy [1]. In the United States, the impact safety performance of guardrail systems is evaluated in accordance with the *American Association of State Highway and Transportation Officials* (AASHTO) *Manual for Assessing Safety Hardware* (MASH) guidelines, with stringent criteria set to assess the crashworthiness and impact behavior of these systems [2,3]. While steel barriers typically exhibit large deflections during vehicle impacts, MASH safety guidelines indicate that vehicles should not penetrate the tested article, implying that guardrails should not sustain major or complete fractures [2]. It has been demonstrated that any localized component failure has the potential to significantly impact the overall performance and crashworthiness of the roadside safety system [4]. Specifically, guardrail steel rupture near the impact or splice locations can result in catastrophic system failure, allowing the errant vehicle to pass through the system [1]. These situations have been highlighted by numerous full-scale crash tests carried out at the Midwest Roadside Safety Facility (MwRSF) at the University of Nebraska-Lincoln [5,6].

**Figure 1:**
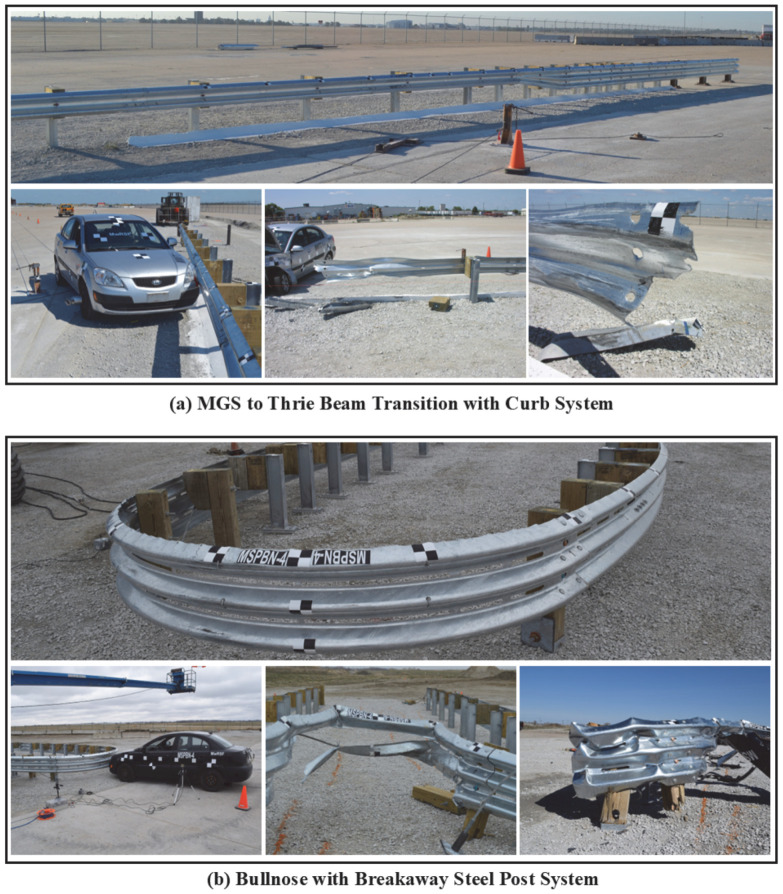
Examples of guardrail steel fractures resulting from full-scale crash testing.

For instance, guardrail steel fracture was observed in a full-scale crash test conducted at the MwRSF in 2012 [5]. In the test, a transition from the W-beam Midwest Guardrail System (MGS) to the thrie beam with a concrete curb was evaluated under MASH test number 3–20. Both W and thrie beam sections were manufactured using AASHTO M-180 guardrail steel. In this case, a small passenger car traveling at 101.2 km/h impacted the system at an angle of 25 degrees. As indicated in Figure 1a, the guardrail barrier failed as the vehicle extended and wedged under the W-beam rail, which resulted in severe system damage that encompassed rail rupture at a splice location. Another example of a guardrail fracture occurred recently when the performance of a Bullnose system was evaluated and crash tested [6]. In this test, a small car impacted the thrie beam Bullnose system at a speed of 99.9 km/h. The system failed to satisfy MASH evaluation criteria, as the vehicle was inadequately contained, and windshield deformation exceeded the limits. As illustrated in Figure 1b, this resulted in significant barrier damage, which consisted of extensive bending and deformation, rail tearing, and disengagement of the breakaway posts.

Given the high cost and time duration required to perform full-scale crash testing, Finite Element Analysis (FEA) has emerged as a viable alternative approach for evaluating the crashworthiness and impact performance of roadside safety hardware, such as W-beam guardrail systems [7]. Consequently, FEA is frequently employed to simulate actual collision scenarios to predict barrier performance before conducting expensive full-scale crash testing. Hence, performing high-fidelity FEA is now considered an indispensable stage prior to conducting crash tests to develop optimal system designs. To guarantee reliable computer simulations, an appropriate FE model should be developed and calibrated to accurately mimic the actual behavior of the system [4]. To achieve optimal accuracy and predictive capabilities of the FEA, choosing an adequate material model and corresponding input parameters becomes vital [8]. However, it has been demonstrated that there is no available FE material model fully developed to accurately predict the fracture behavior of AASHTO M-180 guardrail steel [1,9]. One major reason is that the factors contributing to material failure and the associated failure mechanism are not fully understood [10]. Another valid reason is that conducting comprehensive material characterization studies in conjunction with detailed experimental testing to extract material properties under various loading conditions involves considerable effort, substantial cost, and top-tier facilities. The absence of detailed failure models, specifically those that correlate failure to the state of stress, can lead to inaccurate response prediction, which in turn, jeopardizes safety and potentially results in inadequate designs that fail under real-world crash conditions [11,12,13]. Consequently, alternative models that utilize simplified failure criteria have been frequently used. Nevertheless, these models do not account for damage accumulation or stress-state-dependent failure, which may lead to inconsistent or unreliable material behavior representation.

The concept of the stress state is frequently used to represent the interaction between material particles under the influence of complex loading. It was shown by several researchers that the ductile fracture of a material is governed by the state of stress [14,15,16,17,18]. It has long been recognized that failure under a uniaxial state of stress is not typical for metals, as they tend to fail under two- or three-dimensional states of stress [14,17,18,19]. It has been revealed that ductile fracture of metals is highly dependent on the hydrostatic stress component and the third stress invariant [14,15,16,20]. More specifically, the magnitude of failure strain is found to be highly influenced by two dimensionless parameters, the stress triaxiality and Lode parameter. While the stress triaxiality defines the degree of hydrostatic stress for any given state of stress, the Lode parameter considers the third stress invariant, leading to a three-dimensional failure representation [17,21,22]. Accordingly, widely accepted failure theories and research efforts adopt this failure criterion to incorporate the effect of complex states of stress in predicting ductile failure of metals [13,23,24,25,26].

Considerable research has been devoted to developing an accurate stress-state-dependent ductile failure model. For example, a series of experimental and numerical investigations has been completed by Gao et al. [26] that examines the ductile failure response of DH36 steel plates as a function of stress triaxiality and Lode parameter. Another study carried out by Mohr et al. [4] studied the fracture behavior of Al-7Si-Mg Aluminum alloys. Results from these studies were utilized to develop user-friendly computational material models that can accurately predict fracture behavior. Another study performed by M. Ganjiani investigated the stress-state-dependent fracture performance to propose a FE material model that can be implemented to simulate the response of various metallic alloys under various loading conditions [22]. In the same context, a new ductile failure criterion that accounts for the effects of stress triaxiality and Lode parameter was proposed by Yang et al. [13]. This model was then calibrated against test results of 2024-T351 aluminum alloy, TRIP690 steel, and additively manufactured Ti-6Al-4V. Similarly, numerous research studies have been carried out that examine the fracture of various metals and develop computational constitutive material models [11,19,27,28,29,30,31,32,33,34]. Despite these considerable and extensive efforts, further studies are still needed since the reported findings can only be used to characterize specific materials. Particularly, complete models of ductile failure of many metallic materials, such as AASHTO M-180 guardrail steel, are yet to be constructed. Furthermore, the available material failure models are tailored to encompass loading conditions adequate for sheet forming implementations, which are completely different than those encountered in guardrail steel barriers.

While numerous studies have addressed the ductile fracture behavior of metallic materials using stress-state-dependent criteria [11,13,22,26], to the best of the authors’ knowledge, no published or cited study has focused on the complete material characterization and fracture modeling of AASHTO M-180 guardrail steel under vehicular impact conditions. Thus, this study fills a critical gap by providing experimentally calibrated inputs and stress-state-dependent failure criteria tailored to this widely used roadside safety material. Exploring stress-state-dependent ductile failure is crucial to enhance the capabilities of FE simulations and advance the understanding of failure in AASHTO Specification M-180 guardrail steel. As a result, this study aims to develop and calibrate input parameters for advanced LS-DYNA FE material models to accurately simulate the ductile fracture behavior of AASHTO M-180 guardrail steel under complex, vehicle-induced loading conditions. The derived failure surface and material inputs are experimentally validated and implemented to support predictive finite element simulations of roadside safety hardware. The utilized material model is intended to be simple and flexible enough to accommodate any variation in stress state, avoiding any tedious model tweaking and lengthy trial-and-error procedures. The present paper reports the outcomes from several research project stages that involved extensive experimental and numerical efforts [1]. Research outcomes are expected to be invaluable resources for the roadside safety community as they will enhance the current understanding of guardrail performance and significantly contribute to its practical applications.

## 2. Preliminary Knowledge

The concept of the stress state is of paramount significance in the fields of mechanics and material science as it focuses on the internal forces that particles in a material exert on each other under the influence of external loads. Hence, understanding the state of stress is pivotal in predicting the behavior of a material under various combinations of loading scenarios, and, more importantly, facilitating the prediction of potential failure. In general, a tensor is a mathematical representation of a physical property. In continuum mechanics, the stress tensor (σ) is a fundamental concept that is used to define the state of stress at any point in a body [35]. As expressed in Equation (1), a true stress tensor comprises two distinct components, hydrostatic stress ($\sigma_{H}$) and deviatoric stress (s) [24]. Here, the hydrostatic stress tensor is defined by the product of the trace of a tensor (i.e., $tr\left(\sigma \right)$ the sum of its diagonal components), and ***I***, which is the second-order identity tensor. The hydrostatic stress component represents the isotropic portion of the stress tensor, which corresponds to the uniform pressure experienced by a material [36,37]. Hydrostatic stress is also equivalent to the negative value of the uniform pressure (p) being exerted on the particle of the material. On the other hand, deviatoric stress represents the shear stresses.

(1) $$\sigma =s+\sigma_{H}I=s+\frac{1}{3}tr\left(\sigma \right)I=s-pI$$

It is well known that ductile materials undergo large plastic deformations before failure [13]. These deformations can be attributed to the volumetric and shape changes. In general, hydrostatic stress induces volumetric changes, while deviatoric stress triggers shape deformations [36,37]. For isotropic materials, stress tensors can also be expressed as three stress invariants ($I_{1},\;I_{2},\;I_{3}$) [13]. As shown in Equations (2)–(4), these invariants are utilized to provide other means to describe the state of stress [20,21]. Another basic concept in ductile fracture and mechanics of materials is the von Mises or equivalent stress ($\sigma_{m}$), as represented in Equation (5). In this equation, the operator s:s represents the double contraction of the deviatoric stress tensor with itself, which corresponds to the second invariant of deviatoric stress (i.e., $s:s=\sum_{i,j}s_{ij}^{2}$). Typically, the von Mises stress corresponds to the equivalent shear stress and is commonly used to identify the yielding of a material [38]. It has been indicated that void growth and coalescence, which control the microscopic ductile failure of metals, are mainly influenced by the Von Mises stress ($\sigma_{m}$) [13,38]. The Von Mises stress can also be expressed in terms of the second invariant ($J_{2}$) of the deviatoric stress, which refers to distortional stress resulting from the yielding of an isotropic ductile material.

(2) $$I_{1}=tr(\sigma )$$

(3) $$I_{2}=\frac{1}{2}\left[{\left(tr\left(\sigma \right)\right)}^{2}+tr(\sigma^{2})\right]$$

(4) $$I_{3}=det(\sigma )$$

(5) $$\sigma_{m}=\sqrt{\frac{3}{2}s:s}=\sqrt{3J_{2}}$$

Numerous research efforts have demonstrated that the ductile fracture of metals is highly dependent on the hydrostatic stress component [14,15,16,20]. Initially, Bridgman reported that the ratio between the initial cross-sectional area and the area after fracture at the neck of a round steel bar being tested under uniaxial tension is directly related to the induced confining pressure [14]. In fracture mechanics, stress triaxiality (η) is a dimensionless parameter that is frequently used to identify the degree of hydrostatic stress for any given state of stress [21]. As demonstrated in Equation (6), stress triaxiality is defined as the ratio between hydrostatic stress and von Mises stress, which is an indirect representation of the first and second stress invariants [17,26,35]. Currently, stress triaxiality is widely employed among researchers to investigate the fracture behavior of ductile metals [13,17,20,22].

Although stress triaxiality has been widely recognized as a key factor in the development of ductile damage, numerous research studies have shown that it is not the only determinant, particularly when the material experiences complex loading conditions [13,16,17,22,25,26]. Rather than only using two of the three invariants of the state of stress, information from all three invariants is needed to describe the state of stress in crash and ballistic impact applications. To extend the failure model to capture all the characteristics of the state of stress for an isotropic material, it was indicated that incorporating the third stress invariant as an additional parameter is essential to establish a full characterization of triaxiality [16,17]. It was revealed in these studies that the Lode parameter (ξ), which is commonly referred to as the normalized third deviatoric stress invariant ($J_{3}$), influences the fracture of ductile metals in addition to stress triaxiality and should be taken into account. Lode parameter and the third deviatoric stress invariant are given in Equations (7) and (8). Following relevant previous research studies and given that this study intends to examine the ductile fracture of AASHTO M-180 guardrail steel under impact loading, stress triaxiality and the Lode parameter were employed to establish the stress-state-dependent ductile failure criteria.

(6) $$\eta =\frac{\sigma_{H}}{\sigma_{m}}=-\frac{p}{\sigma_{m}}$$

(7) $$\xi =\frac{27}{2}\frac{J_{3}}{\sigma_{m}}$$

(8) $$J_{3}=det(s)$$

## 3. Experimental Testing and Material Characterization

### 3.1. Material Selection

Given the challenges associated with extracting round and complex shapes of test specimens from standard W-beam rail sections, identifying a substitute material that precisely represents the mechanical and chemical properties of AASHTO M-180 guardrail steel and can be readily processed and formed is pivotal. The AASHTO M-180 steel specification outlines the minimum acceptable mechanical properties, such as yield strength, ultimate tensile strength, and elongation, as detailed in Table 1. A survey of guardrails available in the market was conducted to establish a range of mechanical properties deemed suitable, with material certificates and properties compiled in a recent study by Schmidt et al., facilitating the computation of median, 15th, and 85th percentile values for yield strength, tensile strength, and elongation [39]. This data was instrumental in setting reasonable bounds during the material search. Furthermore, the AASHTO standards do not specify a particular ASTM specification that the guardrail should comply with, other than requirements concerning bolts, nuts, and Zinc coatings. This underscores the critical role of selecting a steel specification that delineates the material required for the component testing program. In accordance with the findings of Schrum et al. [40], it was concluded that ASTM A572 steel can closely resemble AASHTO M-180 guardrail steel available in the market in terms of chemical and mechanical properties, making it a feasible substitute material option for the component testing program [41]. ASTM A572 Grade 50 steel plate of the designated material with 0.5 inches (12.7 mm) thickness and 60 inches (1524 mm) width and with properties comparable to the median values of the range of materials accessible in the market was used, as demonstrated in Table 1. It is worth noting that this selected material falls within the 15th and 85th percentile ranges for both yield and ultimate strength, while demonstrating an elongation that slightly exceeds the 85th percentile, which was deemed acceptable as it falls within the range of compiled information on material available in the market.

### 3.2. Development of Component Testing Program

It has been demonstrated that failure in guardrail steel under vehicle impact predominantly occurs under complex three-dimensional states of stress, characterized by a wide range of stress triaxiality and Lode parameter values that influence ductile fracture behavior [19,26,30]. Typically, the effects of stress state on ductile fracture can be expressed through specially designed experiments that involve standard and non-standard test specimens [26]. Accordingly, the configuration and loading pattern of test specimens must be carefully selected to represent distinct stress triaxiality, Lode parameter, and effective plastic strain at failure [11]. Building upon these insights, a material testing program was developed assuming that the selected material, representing AASHTO M-180 guardrail steel, will fail at an effective plastic strain depending on the state of stress. Following several research studies, 21 test specimens with different geometries were selected and tested under tension, compression, shear, punching, and torsional loading conditions [16,17,20,25,26,42,43].

#### 3.2.1. Tension Specimens

**Figure 2:**
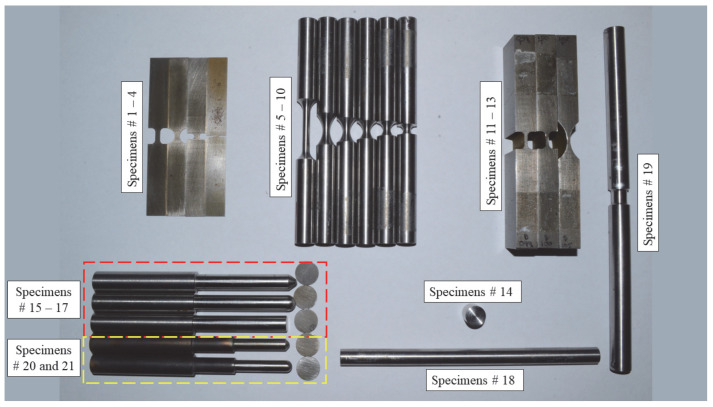
Test specimens.

Thirteen specimen geometries (specimens no. 1 through no. 13), depicted in Figure 2, were tested under tension. The first geometry refers to a flat dog bone specimen, which is a standard specimen geometry that is consistently used to perform uniaxial tension tests. This specimen represents the reference stress state for all other specimens and can be used to extract basic material properties, including elastic modulus and yield strength [1]. The specimen is characterized by η=0.333 and ξ=1. Specimens no. 2 through no. 4 correspond to flat notched geometries that are commonly used to examine the effects of varying stress states associated with plane stress conditions. In this set of specimens, it was indicated that hydrostatic forces developed in the neck region vary with the notch diameter [14]. Particularly, lateral confinement stress increases with reducing the notch diameter, resulting in various stress states along the reduced cross section of the specimen. Thus, these specimens are attributed to η=0.40 to 0.577 and ξ=0 to 1. Similar to specimen no. 1, an axially symmetric, round, smooth specimen is another alternative geometry to perform simple uniaxial tension tests and corresponds to the same set of triaxiality and Lode parameter. Specimens no. 6 through no. 10, identified by notched round geometries, were included in the test program. Again, varying the radius of the notch produces various stress states characterized by η=0.40 to 0.80 and ξ=1. Specimens no. 11 through no. 13 are featured by plane strain conditions, with η=0.577 to 0.80 and ξ=0. In these specimens, thickness and notch size are designed so that the strain in the direction perpendicular to the loading direction is negligible, simulating a plane strain condition. It was indicated that specimens with a thickness that exceeds 25.4 mm can accurately mimic theoretical plane strain conditions when tested under tension [17,25].

#### 3.2.2. Compression and Punch Specimens

Compression and punching test specimens, depicted in Figure 2, were also considered in the test program to provide a diverse range of possible loading scenarios. Specimen no. 14, which refers to the cylindrical upsetting geometry, was tested under uniaxial compression, representing η=−0.333 and ξ=−1. Five punch head shape specimens, associated with specimens nos. 15 through 21, were manufactured and used to test circular thin steel specimens. Punch head shapes and specimen geometries matched those investigated by Buyuk and Seidt and further revised by Humphrey [1,25,43]. Specimens with round and standard heads correspond to η=0.5774 to 0.667 and ξ=−1, while sharp heads are attributed to η=0 and ξ=0.

#### 3.2.3. Shear and Torsion Specimens

Shear and torsion specimens were fabricated and considered in the test program. A Dual-Point Shear Fixture MTS system was employed to test a solid steel rod specimen (specimen no. 18) under shear loading, representing a zero triaxiality and Lode parameter state of stress. A thin hollow tube sample (specimen no. 19) was fabricated and tested under torsion. Both specimen configurations are essentially designed to represent the state of zero triaxiality and Lode parameter.

### 3.3. Testing Setup and Instrumentation

**Figure 3:**
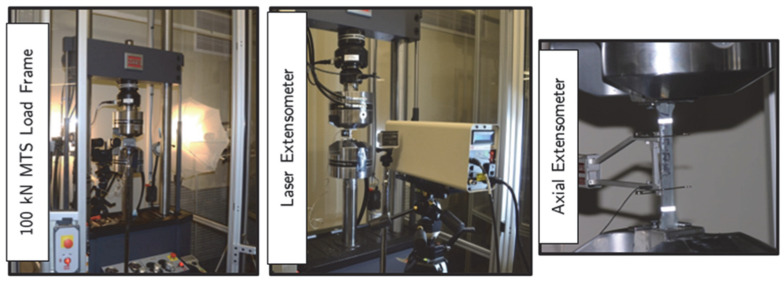
Overview of the tensile test instrumentations and setup.

Tension, compression, shear, and punching tests were completed using two material testing systems (MTS) servo-hydraulic universal testing machines, illustrated in Figure 3. Landmark load frames include a 22-kip (100 kN) fatigue testing frame and a 220-kip (1000 kN) criterion static testing frame. On the other hand, torsion tests were carried out using a Tinius Olsen & Company (Horsham, PA, USA) of Philadelphia torsion frame. Displacements and strains for each specimen were measured using a 1- and 2 in.-gauge length MTS axial extensometer (MTS Systems Corporation, Eden Prairie, MN, USA), the LX 500 laser extensometer (MTS Systems Corporation, Eden Prairie, MN, USA). Following published material testing reports and relevant studies, three-dimensional digital image correlation (3D-DIC) was also employed in the component testing program, as full strain-displacement fields can be accurately determined throughout the specimen loading stages, and more importantly, beyond the necking point [1,25,43]. Additional details on the testing setup and instrumentation can be found elsewhere [1].

### 3.4. Testing Matrix

**Figure 4:**
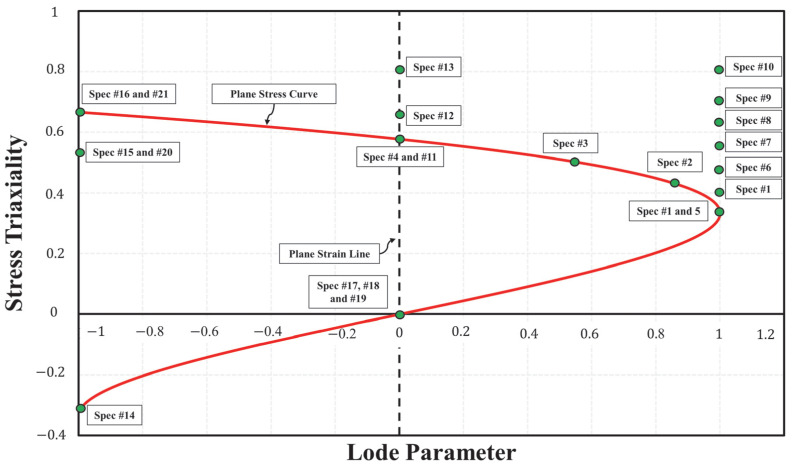
Target stress states of testing specimens.

A total of 124 tests were carried out on the 21 specimens illustrated in Figure 2. While some test results were utilized to extract material properties, the remaining tests were used to provide a diverse range of stress states for evaluating the failure criterion. For instance, the DIC technique was employed to test one standard flat dog bone specimen (specimen no. 1) to identify the elastic modulus and Poisson’s ratio of the tested surrogate material. Five additional tests on specimens no. 1 and no. 5 were conducted using laser and axial extensometers to determine yield strength and the isothermal hardening curve. The complete plot of the targeted stress states, as represented by triaxiality and Lode parameter, for each specimen type is illustrated in Figure 4.

### 3.5. Testing Results

**Figure 5:**
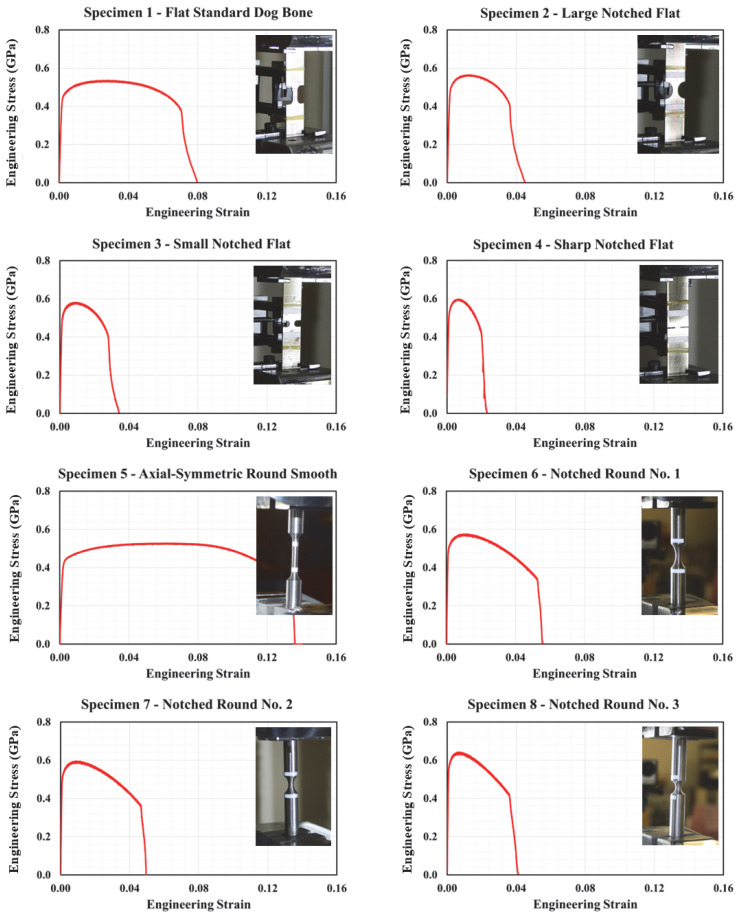
Engineering stress–strain plots for specimens 1–8.

**Figure 6:**
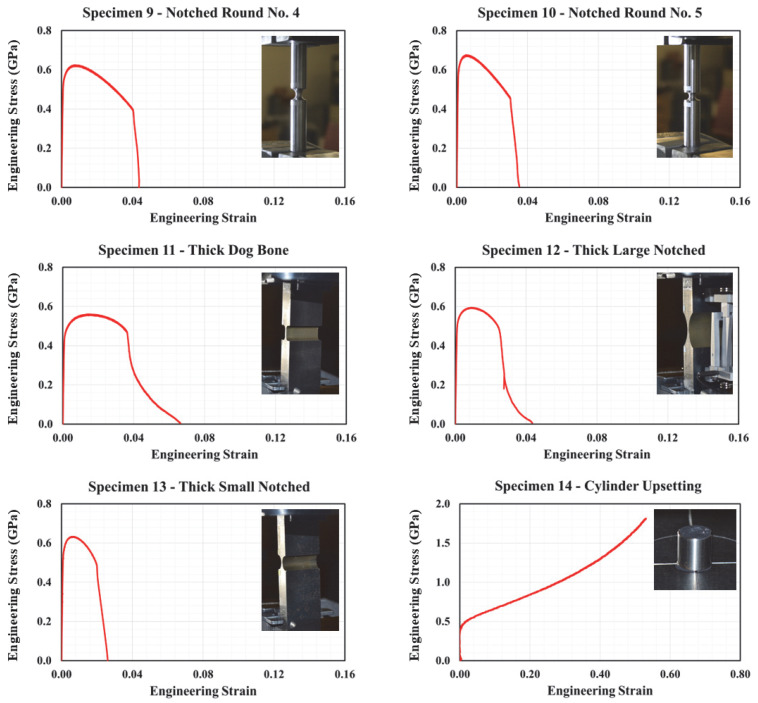
Engineering stress–strain plots for specimens 9–14.

Representative engineering stress–strain curves for specimens no. 1 through no. 14 are illustrated in Figure 5 and Figure 6. It should be noted that at least two distinct tests were performed on each specimen configuration. In each of these tests, slight variations in stress–strain plots were observed. The variation can be attributed to several factors, such as displacement and force measurement methods, and other test uncertainties. Consequently, the plots depicted in these figures correspond to the curves lying between the upper and lower bounding curves to provide a more realistic representation of stress–strain relationships.

**Figure 7:**
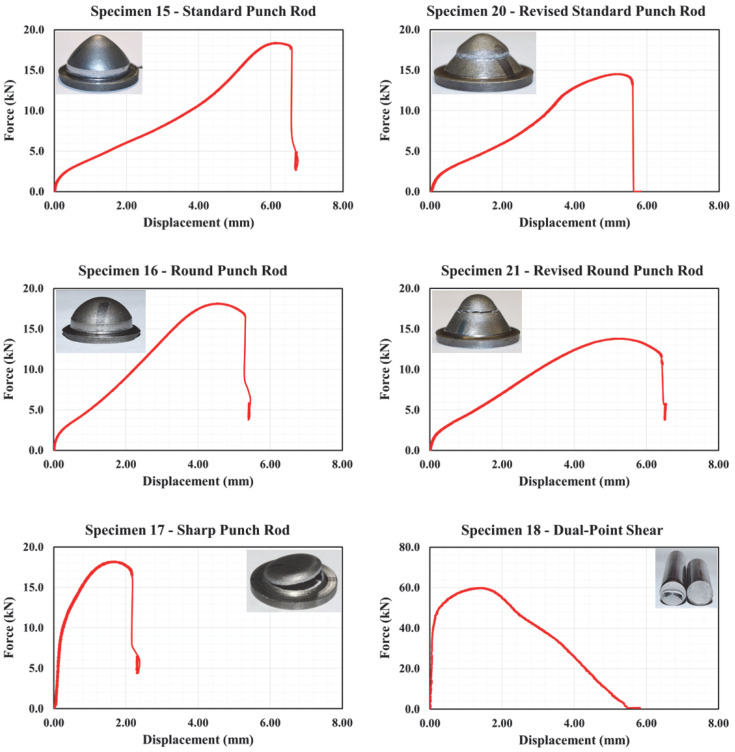
Force-displacement plots for specimens 15–18, 20, and 21.

As shown in Figure 5, specimen no. 1 demonstrated typical ductile behavior characterized by an initial elastic region followed by substantial plastic deformation. As expected and due to stress concentration at the notch, specimens no. 2 through no. 4 demonstrated lower failure strain values compared to specimen no. 1. In particular, reducing the notch size contributed to increasing stress triaxiality, which resulted in promoting localized deformation and decreasing the overall ductility. Similar to specimen no. 1, the axially symmetric, round, smooth specimen (specimen no. 5) experienced consistent stress distribution with high ductility. Again, the influence of altering notch size and the corresponding higher levels of triaxiality is further highlighted in the stress–strain response of specimens no. 6 through no. 10, where reduced d levels of ductility were observed. Specimens no. 11 through no. 13, which induce a complex state of stress simulating plane strain loading conditions, demonstrated higher resistance to necking due to their relatively larger cross-sectional areas, and thus, high ultimate strengths. While specimen 11 sustained a relatively extended plastic deformation range before failure, the two other specimens demonstrated reduced ductility similar to the flat and round notched specimens. When tested under compression, specimen no. 14 has shown a different failure mechanism, which is characterized by barreling and ultimately shear failure. As shown in Figure 6, the resulting stress significantly increased prior to failure due to the lateral confinement effects. Results from punch and shear tests are illustrated in Figure 7. This figure clearly illustrates the effect of punch head shape on specimen capacity and failure strain. Due to unforeseen technical errors during testing, the force-displacement plot corresponding to specimen no. 19 is not reported herein. A comprehensive summary of component testing results is provided in Table 2 and Table 3.

### 3.6. Elastic and Plastic Flow Parameters Extraction

Accurately extracting elastic and plastic material properties from experimental testing and adopting an appropriate elastoplastic material model are the primary steps to guarantee reliable simulations of guardrail steel behavior, specifically the fracture mechanism [19]. Uniaxial tensile tests carried out on standard tensile specimens, which are fabricated per universal specifications, such as ASTM E8, are typically used to identify the mechanical properties of metals [41,44]. Tension tests are commonly used to identify major material parameters, including yield strength ($\sigma_{y}$), ultimate tensile strength ($\sigma_{u}$), elastic modulus (E), Poisson’s ratio (ν), and failure strain ($\varepsilon_{f}$). These parameters are evaluated through establishing a constitutive relationship between the applied tensile load and the specimen’s deformation, which is commonly expressed in the form of force-displacement or stress–strain curves. In this study, tests carried out on specimen no. 1, which represented uniaxial tension, were used to identify the elastic material properties. The three-dimensional digital image correlation (3D-DIC) technique was employed to accurately measure variations in the axial and transverse strains. Accordingly, the elastic modulus and Poisson’s ratio were found to be 199.3 GPa and 0.295, respectively. While these properties are essential for simulating the material’s initial elastic response and response during unloading, post-yielding, plastic behavior requires characterization of the flow parameters. Engineering and true stress–strain relationships are widely accepted among researchers to represent the behavior of metals [35]. Engineering stress–strain curves correspond to the initial undeformed configuration when calculating stress from load. In uniaxial extension, engineering stress (σ) and engineering strain (ε) are expressed by Equations (9) and (10), where F is the applied tensile force, $A_{o}$ is the initial cross-sectional area, and $l_{o}$ and $l_{f}$ are the initial and final gauge lengths, respectively.

(9) $$\sigma =\frac{F}{A_{o}}$$

(10) $$\varepsilon =\frac{l_{f}-l_{o}}{l_{o}}$$

While engineering stress–strain curves is useful for initial material characterization, they become less accurate when a material undergoes large deformations. This is because they do not account for the reduction in cross-sectional areas and the actual local elongation of the specimen, as they assume constant cross-sectional area and uniform elongation throughout the test. Accordingly, utilizing alternative stress–strain measurements is pivotal to provide a more accurate representation of local material response. Hence, the true stress–strain relationship is frequently utilized as it considers an instantaneous cross-sectional area. True stress–strain curves are subsequently adopted in numerous implementations that involve large plastic deformations, such as metal forming and roadside safety applications. True stress and true strain are given in Equations (11) and (12). It is noteworthy to mention that engineering and true stress–strain curves are close to the yielding point, and the discrepancy significantly increases as the material experiences large plastic deformations. Once the diffused necking begins, these equations are no longer applicable. This is mainly because the uniaxial state of stress is transformed to a triaxial stress state, leading to nonuniform plastic deformation [44,45].

(11) $$\sigma_{T}=\sigma \;(1+\varepsilon )$$

(12) $$\varepsilon_{T}=\ln\left(1+\varepsilon \right)$$

Failure strain can be calculated using the adjusted method proposed by Bridgman [14], as illustrated in Equation (13), where $A_{i}$ and $A_{f}$ are the initial and final cross-sectional areas, respectively. On the other hand, diffused necking occurs when the true stress is equal to its hardening rate, which refers to Considère’s necking criterion, as shown in Equation (14) [19]. Graphically, the necking stress is located at the intersection between the true stress–strain curve and its derivative.

(13) $$\varepsilon_{F}=\ln\left(\frac{A_{i}}{A_{f}}\right)$$

(14) $$\sigma_{T}=\frac{d\sigma_{T}}{d\varepsilon_{T}}$$

In FE simulations, the input of true stress–strain data that precisely represents the plastic flow of a material is essential to ensure the accuracy of the results. Although linear interpolation is frequently used to define post-necking true stress–strain behavior, the process is quite tedious, as it involves several trials to achieve an adequate match between simulation and test results. As the ultimate goal of the current study is to provide consistent material characteristics that can be consistently adopted to model AASHTO M-180 guard rail steel, an alternative and more feasible approach to characterize the post necking behavior was implemented. Moreover, the selected strain hardening model should be robust and stable when various specimen configurations are numerically investigated. Numerous mathematical models to estimate post-necking true stress–strain are available in the open literature [46]. Following a preliminary FE simulation stage, the Swift model, which is extensively used to model isotropic hardening of metals, was adopted in the present study to represent the material’s plastic flow [47]. The model can be used to express the true stress as a function of true (or effective) plastic strain ($\varepsilon_{P}$), which can be obtained by subtracting the elastic strain portion from the total strain value, as expressed in Equation (15). The Swift model is provided in Equation (16), where K, $\varepsilon_{o}$, and n are material constants.

(15) $$\varepsilon_{p}=\varepsilon_{T}-\frac{\sigma_{T}}{E}$$

(16) $$\sigma_{T}=K\;{\left(\varepsilon_{o}+\varepsilon_{p}\right)}^{n}$$

**Figure 8:**
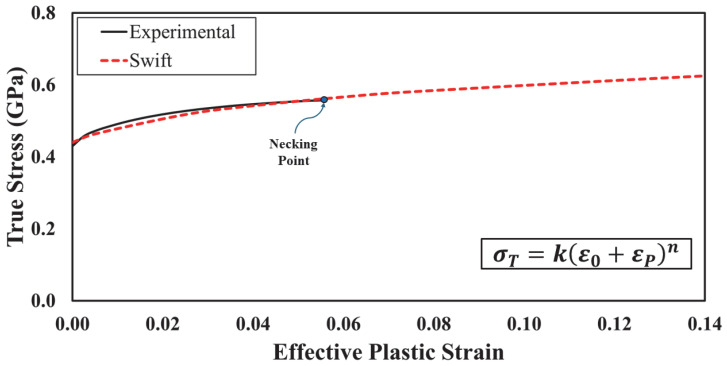
Derived plasticity curve.

Initially, the true stress–strain response associated with specimen no. 5 was extracted. 3D-DIC was employed to estimate the true axial strain and the subsequent effective plastic strain. Non-linear regression analysis was implemented, using IBM SPSS software version 25, to fit the Swift model with the measured true stress–strain data up to the necking point. Following multiple iterations, optimized Swift model parameters were found to be K = 0.8 GPa, $\varepsilon_{o}$ = 0.01, and n = 0.13. The final Swift model was then used to define the full relationship between true stress and effective plastic strain (i.e., beyond necking and until failure). The final proposed plasticity curve is shown in Figure 8.

## 4. Finite Element Analysis

### 4.1. Material Models

This section presents a brief overview of the three selected LS-DYNA 14.0 material models commonly used to model ductile failure. These models were selected due to popularity and proven effectiveness in simulating material responses in crashworthiness applications.

#### 4.1.1. Tabulated Johnson–Cook Model

An extensive collaborative effort involving the Federal Aviation Administration (FAA), National Aeronautics and Space Administration (NASA), George Washington University (GWU), Ohio State University (OSU), and George Mason University (GMU) has led to the development of the Tabulated Johnson–Cook (*MAT224*) constitutive material model in LS-DYNA 14.0 for simulating metals [48]. This model has been recognized by several researchers as a viable tool for various practical applications, including aerospace and roadside safety applications [1,48]. The Tabulated Johnson–Cook model builds on the existing Johnson–Cook material model (*MAT15*) in LS-DYNA 14.0, with the added capability of incorporating tabulated input parameters. Beyond defining the material’s density and elastic properties, this elastic thermo-viscoplastic material model allows for the input of multiple stress–strain curves under varying strain rates and temperatures, as expressed in Equation (17) [49]. In this equation, σ is the flow stress, A is the yield stress at a reference temperature and strain rate, B is the coefficient of strain hardening, n is the strain hardening exponent, $\varepsilon^{p}$ is the effective plastic strain, C and m are material constants, $T^{*}$ is the homologous temperature, which is given by Equation (18) and relates the material’s temperature to the room and melting temperatures.

(17) $$\sigma =\left(A+B{\varepsilon^{p}}^{n}\right)(1+C\ln{\dot{\varepsilon }}^{p})(1-{T^{*}}^{m})$$

(18) $$T^{*}=\frac{T-T_{room}}{T_{melt}-T_{room}}$$

In *MAT224*, the effective plastic failure strain (${\varepsilon^{P}}_{f}$) is defined as a function of several critical parameters: stress triaxiality (η), Lode parameter (ξ), total or plastic strain rates ($\dot{\varepsilon }\;$ or ${\dot{\varepsilon }}^{p}$), temperature (T), and element size ($l_{o}$) [1]. It is important to note that failure strain can be specified as a function of one or more of these parameters. When multiple parameters are employed, the failure strain is calculated as the product of these functions, as shown in Equation (19). To accurately model stress-state-dependent failure, the latest version of LS-DYNA 14.0 enables the input of a curve that defines the effective failure strain as a function of stress triaxiality. Additionally, for solid elements, a table of curves, which is a failure surface, can be utilized to relate the failure strain to both stress triaxiality and Lode parameter. The load curve or table ID can be specified using the *LCF* variable on the second input card. Furthermore, the *LCG* and *LCH* variables are used to assign load curve IDs, where each curve defines failure strain as a function of strain rate or temperature. A specific curve or table ID can be entered under *LCI* to correlate the failure strain to element size and stress triaxiality. It is noteworthy that the stress triaxiality referenced in all input curves corresponds to the ratio of pressure (i.e., $-\sigma_{H}$) to von Mises stress.

(19) $${\varepsilon^{P}}_{f}=f\left(\eta ,\;\xi \right)g\left({\dot{\varepsilon }}^{p}\right)h\left(T\right)i(\eta ,l_{o})$$

The Tabulated Johnson–Cook material model incorporates two built-in failure criteria. The default failure criterion is based on the accumulation over time of the effective plastic strain rate over the effective failure strain, as shown in Equation (20). This approach accounts for load-path dependent failure. For modeling load-path independent failure, an alternative criterion can be activated (*FAILOPT* = 1.0), as outlined in Equation (21), where only the current state of plastic strain is evaluated. When this criterion is enabled, the effective failure plastic strain is averaged over several time steps, which can be controlled using the *NUMAVG* variable. Element deletion occurs when either F or $F_{2}$ is reached or exceeded unity.

(20) $$F=\int \frac{{\dot{\varepsilon }}^{P}}{{\varepsilon^{P}}_{f}}\;dt$$

(21) $$F_{2}=\frac{\varepsilon^{P}}{{\varepsilon^{P}}_{f}}$$

#### 4.1.2. Generalized Incremental Stress-State-Dependent Model (GISSMO)

Several LS-DYNA 14.0 material models have been developed to address material damage and failure [50]. Many of these models combine elastoplasticity with damage formulations and embedded failure criteria, such as the Johnson–Cook models (*MAT15* and *MAT224*). On the other hand, LS-DYNA 14.0 supports the definition of damage and failure through the *MAT_ADD_EROSION keyword [50]. This keyword allows users to specify a failure model, enabling the integration of distinct failure criteria with the chosen material model. One frequently used model for predicting failure based on damage accumulation is the Generalized Incremental Stress-State-Dependent Model (*GISSMO*), which can be implemented via the *MAT_ADD_EROSION* keyword [23]. Originally developed by Neukamm and Haufe in 2010 and further refined by Andrade in 2016 for crashworthiness applications, *GISSMO* provides a robust framework for simulating material failure [24,51,52]. Notably, LS-DYNA also offers the *MAT_ADD_DAMAGE_GISSMO* keyword, a variant of the *MAT_ADD_EROSION* keyword, which facilitates the direct implementation of the *GISSMO* failure model. When *GISSMO* is incorporated, damage accumulation (∆D) is calculated at each time step according to Equation (22). In this equation, *DMGEXP* refers to the damage exponent, $\varepsilon_{f}(\eta ,\xi )$ is the stress state dependent failure strain (i.e., function of the stress triaxiality and Lode parameter), *D* is the current damage value, and $\Delta \varepsilon_{p}$ is the equivalent plastic strain increment [49]. Similar to *MAT224*, a load curve ID that defines failure strain as a function of stress triaxiality, or a table ID that accounts for the effects of stress triaxiality and the Lode parameter, can be specified using the *LCSDG* variable [49]. Unlike *MAT224*, stress triaxiality is defined according to Equation (6), where hydrostatic stress is divided by the von Mises stress. Element deletion is triggered when the damage value of D reaches or exceeds unity.

(22) $$∆D=\frac{DMGEXP}{\varepsilon_{f}(\eta ,\xi )}D^{\left(\frac{DMGEXP-1}{DMGEXP}\right)}\Delta \varepsilon_{p}$$

Through the *GISSMO* model, an additional parameter, *F*, which serves as an instability measure defining the onset of diffused necking, can be activated [53]. When this instability variable is used, coupling between damage and stress flow will be initiated and evaluated at each time step, as shown in Equation (23) [24]. In this context, $\varepsilon_{crit}(\eta ,\xi )$ represents the critical strain at the onset of diffused necking and can be defined as a function of stress-state variable $\varepsilon_{f}(\eta ,\xi )$. The undeformed material is characterized by *F* = 0, while *F* = 1 indicates the initiation of damage localization. Upon reaching *F* = 1, the current damage value is set to the critical damage value ($D_{crit}$) [24,52]. Beyond this point, the flow stress and damage are coupled as per Equation (24), where $\tilde{\sigma }$ is the undamaged stress tensor, and m is the fade exponent, which governs the rate of energy dissipation and stress decay and is typically calibrated to align with experimental results [23,54]. The model also offers two alternative methods for defining the coupling between flow stress and damage. The first approach involves specifying a constant critical damage value ($D_{crit}$). In this case, coupling is initiated when the current damage value (D) surpasses $D_{crit}$. The second approach sets a fixed value for the critical plastic strain. Once the plastic strain reaches this predefined value, the current damage is stored as $D_{crit}$, thereby activating stress-damage coupling [49].

(23) $$∆F=\frac{DMGEXP}{\varepsilon_{crit}(\eta ,\xi )}F^{\left(\frac{DMGEXP-1}{DMGEXP}\right)}\Delta \varepsilon_{p}$$

(24) $$\sigma =\tilde{\sigma }\left[1-{\left(\frac{D-D_{crit}}{1-D_{crit}}\right)}^{m}\right]$$

#### 4.1.3. Piecewise-Linear Plasticity Model

The *Piecewise Linear Plasticity* material model (*MAT24*) in LS-DYNA 14.0 is frequently used in crashworthiness applications [1,49]. Despite the simplified failure criterion, which is a constant effective plastic strain at failure, MAT24 offers several benefits over other material models, such as its computational-cost efficiency and strain-rate dependency [35]. This material model is based on elasto-plasticity and handles strain-rate effects using the Cowper–Symonds strain-rate model, and includes the von Mises yield criterion, the associated flow rule, and isotropic strain hardening. In this model, the deviator stress is determined to satisfy the yield function as provided in Equation (25), where $s_{ij}$ are the components of the deviatoric stress tensor. Yield stress, $\sigma_{y}$ is expressed in Equation (26). In this equation, β is a strain rate factor and accounts for strain-rate effects, $\sigma_{0}$ is the initial yield stress, $f_{h}\left(\varepsilon_{eff}^{p}\right)$ is the hardening function, and can be specified in tabular form or linear hardening of the form $f_{h}\left(\varepsilon_{eff}^{p}\right)=E_{P}\varepsilon_{eff}^{p}$ with plastic hardening modulus $E_{P}$.

(25) $$f=\frac{1}{2}s_{ij}s_{ij}-{\left(\frac{\sigma_{y}}{\sqrt{3}}\right)}^{2}\leq 0$$

(26) $$\sigma_{y}=\beta \left[\sigma_{0}+f_{h}\left(\varepsilon_{eff}^{p}\right)\right]$$

The deviatoric stress is updated elastically, and the yield function is checked. The deviatoric stress is accepted if the yield function is satisfied. Otherwise, the plastic strain increment is calculated as given in Equation (27), where $E_{P}$ is the current hardening modulus, and G  is the shear modulus. The trial deviatoric stress state, ${\tilde{s}}_{ij}$ is scaled back as expressed in Equation (28).

(27) $$\Delta \varepsilon_{eff}^{p}=\frac{{\left(\frac{3}{2}{\tilde{s}}_{ij}{\tilde{s}}_{ij}\right)}^{\frac{1}{2}}-\sigma_{y}}{E_{P}+3G}$$

(28) $$s_{ij}^{n+1}=\frac{\sigma_{y}}{{\left(\frac{3}{2}{\tilde{s}}_{kl}{\tilde{s}}_{kl}\right)}^{\frac{1}{2}}}{\tilde{s}}_{ij}$$

The Cowper–Symonds model [55] scales the yield stress using a factor β, which is calculated following Equation (29), in which, ${\dot{\varepsilon }}_{p}$ is the effective plastic strain rate, and c and p are Cowper–Symonds strain rate parameters.

(29) $$\beta =1+{\left(\frac{{\dot{\varepsilon }}_{p}}{c}\right)}^{\frac{1}{p}}$$

### 4.2. Development of Stress-State-Dependent Failure Surface

**Figure 9:**
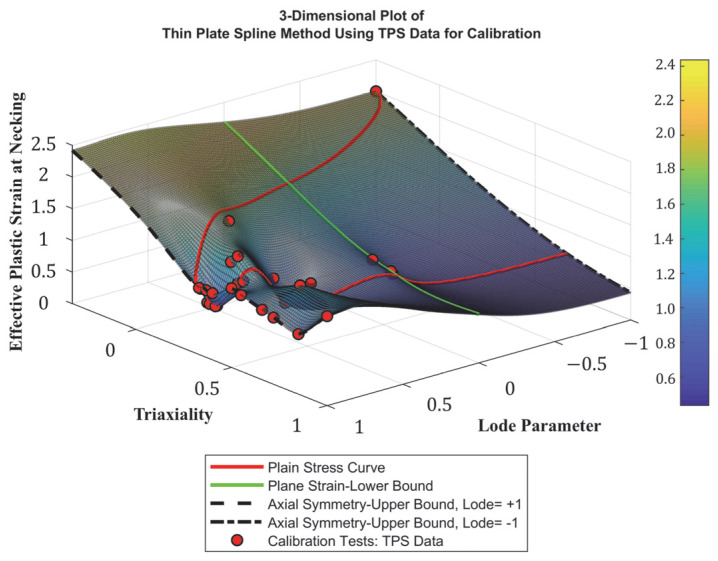
Finalized stress-state-dependent failure surface.

**Figure 10:**
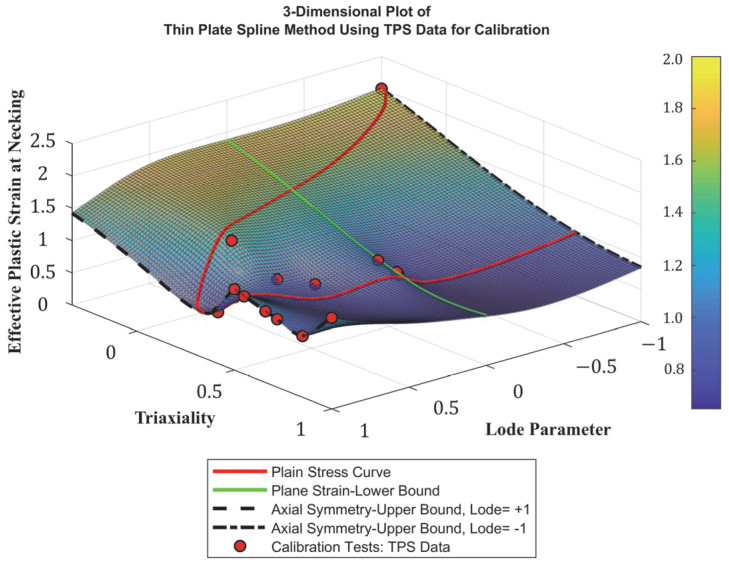
Critical stress-state-dependent surface.

In the context of this paper and other related studies, failure surface refers to the three-dimensional representation of failure strain as a function of stress triaxiality and Lode parameter. Extensive research has been carried out to develop stress-state-dependent failure surfaces that can be utilized in finite element modeling to simulate the fracture behavior of metals [16,18,20,25,56]. Smoothed thin-plate spline (TPS), which is a mathematical interpolation technique used to fit smooth surfaces to a scattered dataset, was employed to establish a baseline failure surface utilizing test results reported by Buyak on Al2024-T351 aluminum, assuming similar response and fracture behavior [1,25,57]. Following a series of FE simulations, and as expected, this failure surface was unable to accurately capture failure under various loading conditions. Hence, the surface was modified to fit the test results summarized previously. In these tests, each specimen configuration is attributed to a unique stress state (i.e., η and ξ) and failure strain value, which is represented a single point on the failure surface. Despite being theoretically possible, obtaining every data point of stress triaxiality and Lode parameter to develop a complete failure surface is practically unachievable due to extensive and complex testing requirements. Although utilizing 21 specimens to construct the failure surface was deemed adequate compared to relevant research studies, additional numerical simulations were carried out to provide a more accurate and comprehensive representation of ductile failure [18,25,43,58]. In these simulations, test specimens were modeled, and the *MAT24* and *GISSMO* models were adopted to simulate their response. The failure surface was then tweaked to achieve a reasonable match between numerical and test results. LS-DYNA’s *CONTROL_DEBUG keyword was implemented to extract the stress-state parameters and the corresponding failure strain required to modify the failure surface for each simulated specimen. Consequently, the final optimized failure surface is depicted in Figure 9. As described earlier, the *GISSMO* model enables the users to define critical strain, which is the strain at the onset of diffused necking, as a function of stress triaxiality and Lode parameter. This secondary surface was also developed following the same approach, as illustrated in Figure 10.

### 4.3. FE Models Development

A series of finite element simulations was carried out using LS-DYNA 14.0 to demonstrate the capability of the identified material properties and the stress-state-dependent failure surface in replicating test results. These material characteristics were used to define all inputs for the previously presented three material models (*MAT24*, *MAT224*, and *GISSMO*). In addition to material models, FE simulations considered various model parameters, such as element types and formulations. Comparisons between simulation and test results are presented in this section.

**Figure 11:**
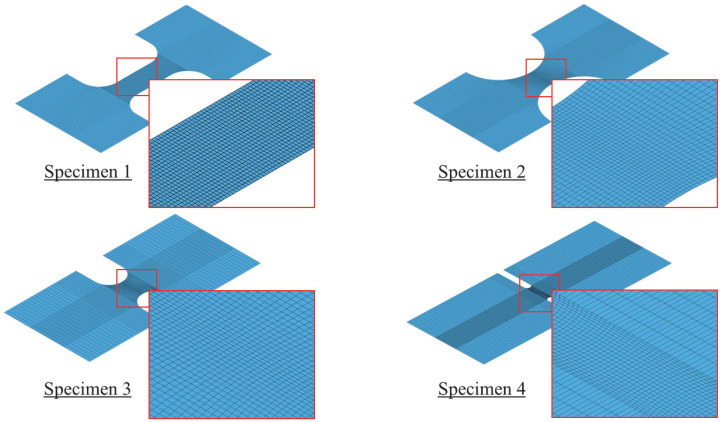
FE models of specimens 1 to 4 meshed using shell elements.

**Figure 12:**
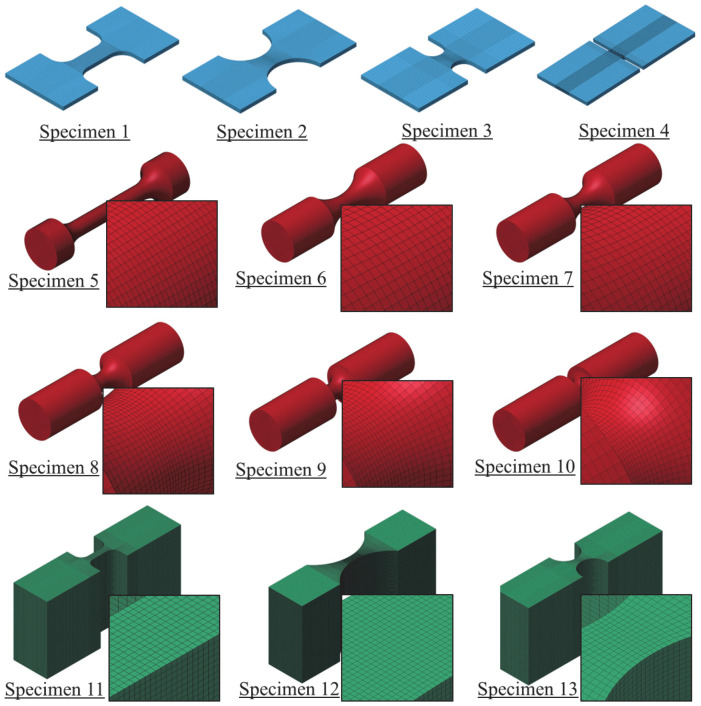
FE models of specimens 1 to 13 meshed using solid elements.

Tension test specimens no. 1 through no. 13 were modeled and simulated using shell and solid elements. Extensive mesh sensitivity analyses were initially completed, and more details can be found elsewhere [1,40]. According to these studies, hexahedral solid elements and quadrilateral shell elements were employed to model the test specimens. It was determined that limiting element size to 0.152 mm or less in the vicinity of the necking region would provide accurate results. Modeled specimens are illustrated in Figure 11 and Figure 12. To examine the robustness of the developed models, under-integrated (*UI*) and fully-integrated (*FI*) quadrilateral shell elements were initially employed to model specimens no. 1 through no. 4. For the *UI* shell elements, the default Belytschko–Tsay (*ELFORM* = 2) was used. *FI* shell elements were modeled using the thickness-enhanced shell element formulation (*ELFORM* = 26) with 2×2 integration points. The shell thickness update option was activated, which accounts for the effects of shell membrane thinning, by setting *ISTUPD* to 1 in the *Control_Shell* keyword. To counterbalance zero energy distortion modes, the standard LS-DYNA’s viscous hourglass control form (*IHQ* = 1) with an hourglass coefficient (*QH*) of 0.01. Similar to shell elements, *UI* and *FI* solid elements were utilized to model all test specimens. The default constant stress solid elements in LS-DYNA 14.0 (*ELFORM* = 1) with single integration point and type 6 hourglass control with a coefficient value of 0.01 were used. For *FI* solid elements, an improved and efficient 8-point hexahedral solid element formulation (*ELFORM* = −1) was used.

Following the model construction, the previously discussed material models, *MAT224* and *MAT24*, were used with and without *GISSMO*. In the present study, *MAT24* was examined when used individually or coupled with the *GISSMO* damage model to allow for stress-state-dependent failure criteria to be defined. Consequently, the robustness of the derived material characteristics was investigated through the implementation of four modeling scenarios: (i) *MAT224* only; (ii) *MAT224* with *GISSMO*; (iii) *MAT24* only; and (iv) *MAT24* with *GISSMO*. In the case that involved *GISSMO*, density, elastic modulus, Poisson’s ratio, yield strength, and plasticity curve were defined within *MAT24* and *MAT224* models, while the failure surface and the damage parameters presented earlier were specified within *ADD_DAMAGE_GISSMO* control cards. Basic material properties and the derived plasticity curve are provided in Table 4 and Table 5, respectively. When *MAT224* was solely used, the failure surface was used as an input in conjunction with the previously mentioned material parameters. For the case in which only *MAT24* was employed, the plastic failure strain (*FAIL*) was set to 1.40 to account for element erosion.

## 5. FE Simulation Results

**Figure 13:**
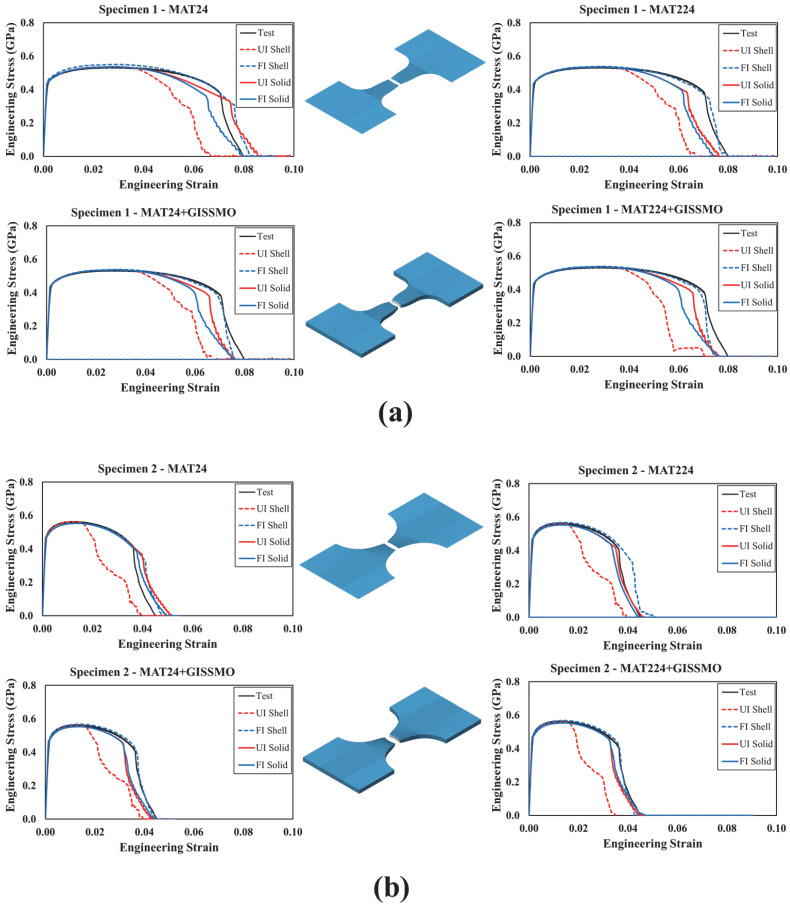
Simulated and tested engineering stress–strain curves: (**a**) specimen 1; (**b**) specimen 2.

**Figure 14:**
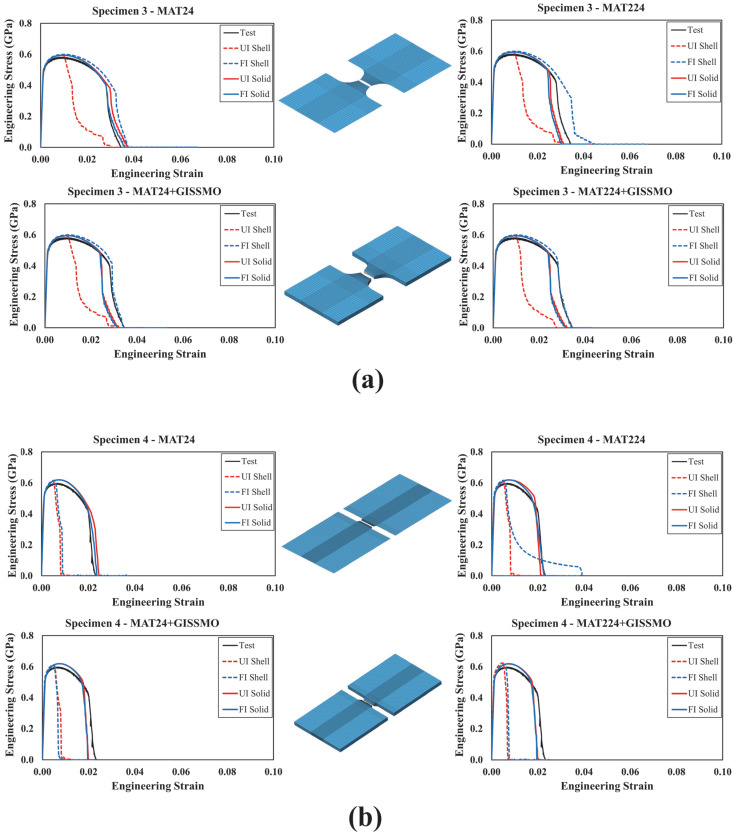
Simulated and tested engineering stress–strain curves: (**a**) specimen 3; (**b**) specimen 4.

**Figure 15:**
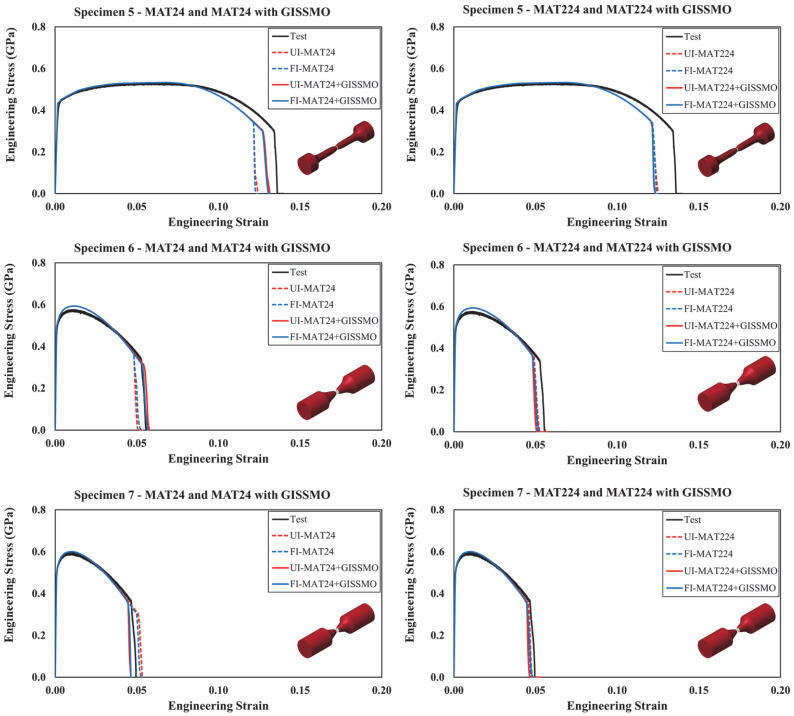
Simulated and tested engineering stress–strain curves: specimens 5 to 7.

**Figure 16:**
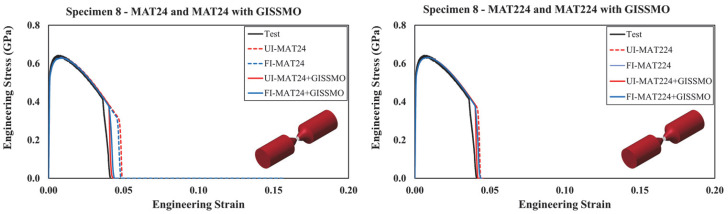

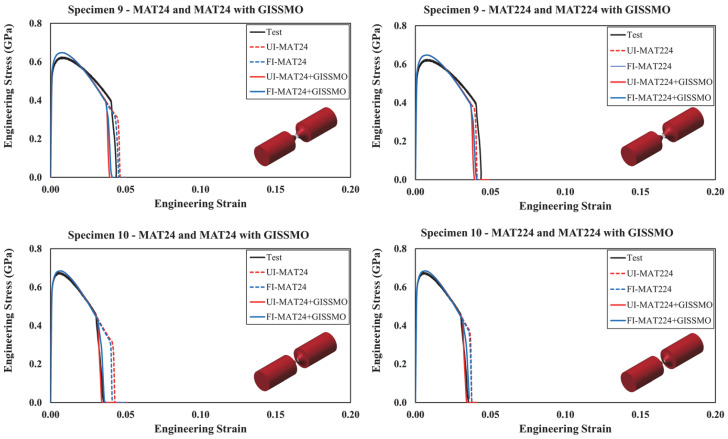
Simulated and tested engineering stress–strain curves: specimens 8 to 10.

**Figure 17:**
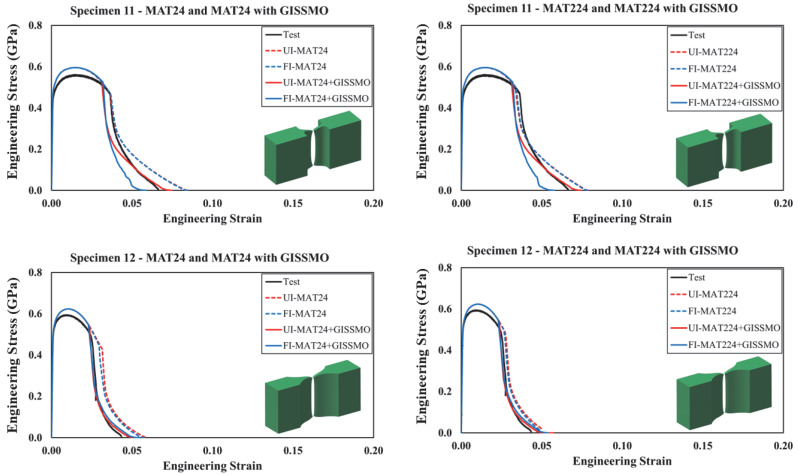

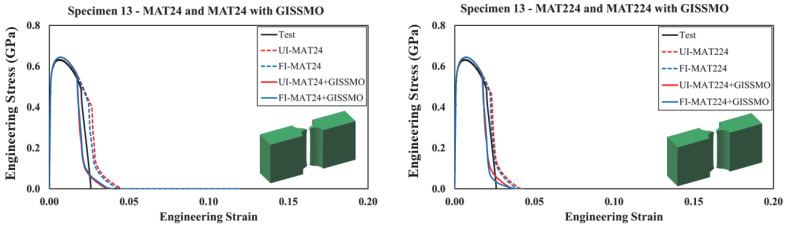
Simulated and tested engineering stress–strain curves: specimens 11 to 13.

In this section, simulated and actual engineering stress–strain curves were compared for all modeled test specimens, as shown in Figure 13, Figure 14, Figure 15, Figure 16 and Figure 17. In Figure 13 and Figure 14, each curve represents a particular material modeling scenario when shell and solid elements were utilized. Again, all material models were explored using *UI* and *FI* element formulations. Irrespective of the material model, it was noted that specimens modeled using *UI* shell elements were not able to accurately reproduce test results. On the other hand, the *FI* shell elements demonstrated good agreement with test results for specimens 1 to 3. However, both *UI* and *FI* shell elements showed a noticeable deviation from the test results of specimen 4. This can be attributed to the complex stress state that this particular specimen configuration encompasses. As described earlier, specimen no. 4 corresponds to a plane strain loading condition characterized by ξ=0, which requires a three-dimensional element representation to account for out-of-plane stresses. This was confirmed when a better correlation between the actual and simulated curves was achieved when solid elements were used, with no significant variation between *UI* and *FI* element formulations. Furthermore, it was obvious that using *GISSMO* with the selected material models enhances the prediction capability of the developed FE models, specifically, under complex loading conditions.

Results from specimens no. 5 to no. 10 are illustrated in Figure 15 and Figure 16. Again, integrating the *GISSMO* damage model with *MAT24* contributed to better results, demonstrating enhanced prediction capability. On the other hand, *MAT224* performed largely the same with and without coupling it with *GISSMO*. However, any slight discrepancy between the modeling approaches can be attributed to the coupling between flow stress and damage uniquely supported by *GISSMO*. For any given material modeling scenario, the response of specimens modeled using *UI* and *FI* solid elements demonstrated good agreement. Accordingly, *UI* solid elements are recommended given their considerably lower computational cost.

Similarly, the proposed material modeling approach is deemed efficient for the response of the more complex specimens (i.e., nos. 11–13), as highlighted in Figure 17. Following the previous observations, incorporating the *GISSMO* damage model into *MAT24* and *MAT224* resulted in improved response and failure prediction, with a more prominent enhancement when *MAT24* was implemented. Particularly, it was observed that using the *GISSMO* damage model improved the capability of the FE models in predicting the most complex loading scenarios attributed to specimens no. 11 through no. 13. Although the ultimate strength is slightly overestimated in specimens no. 11 and no. 12, the overall response of these test specimens is precisely reproduced. As this study intends to avoid a tedious model refinement process by providing a simplified modeling approach and all necessary material model parameters needed to readily simulate the behavior of guardrail steel, no further model tweaking was performed.

**Figure 18:**
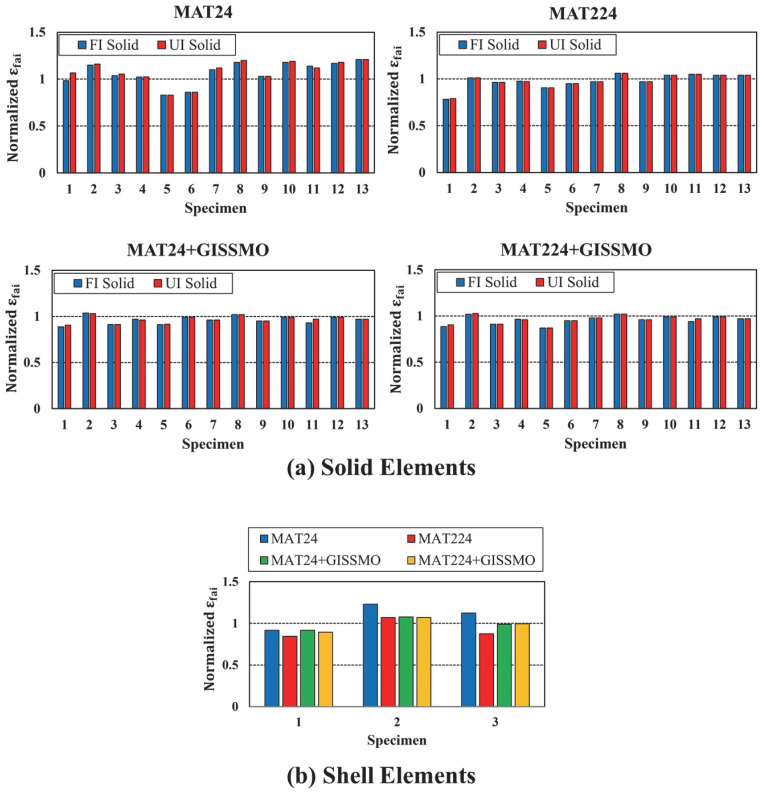
Normalized failure strain, all specimen configurations and material models: (**a**) solid elements; (**b**) shell elements.

To further evaluate the proposed material modeling approach, failure strains predicted by the FE simulations were compared to those obtained from testing, with simulated failure strains referring to the first failed element. Each strain value was then normalized to the corresponding actual failure strain, as shown in Figure 18. This figure emphasizes the accuracy of the derived input parameters and the capability of the selected modeling scenarios to predict the actual failure strain. For solid elements depicted in Figure 18a, it was emphasized that *MAT24* and *MAT224* material models experienced better prediction accuracy when coupled with *GISSMO*. Generally, using *MAT24* only resulted in overestimated failure strain values. On the other hand, using *MAT224* is attributed to relatively better predictions. This is mainly because *MAT224* utilizes stress-state-dependent failure surface to define ductile fracture, which is largely the same approach available in the *GISSMO* damage model. It was also noted that *FI* and *UI* solid element formulations largely behaved similarly. For shell elements illustrated by Figure 18b, only *FI* element formulations were considered in this evaluation, as they demonstrated superior performance compared to *UI* elements. Furthermore, specimen 4 was excluded from this assessment due to the poor performance associated with using shell elements. Again, *MAT24* and *MAT224* integrated with *GISSMO* experienced superior performance.

## 6. Summary and Conclusions

This study presents a comprehensive framework for developing stress-state-dependent failure criteria and calibrated material models aimed at improving finite element predictions of ductile fracture in AASHTO M-180 guardrail steel. The work represents a significant advancement in modeling guardrail behavior for roadside safety applications, particularly through the integration of damage mechanics into LS-DYNA 14.0 simulations.

Due to fabrication limitations associated with W-beam sections, experimental testing was conducted using ASTM A572 Grade 50 steel, selected for its mechanical similarity to AASHTO M-180. Twenty-one uniquely designed specimens were tested under various loading conditions to span a broad range of stress triaxiality (η) and Lode parameter (ξ), enabling the development of a stress-state-dependent failure surface. This surface relates equivalent plastic failure strain (${\varepsilon^{P}}_{f}$) to η and ξ, forming the foundation for material input calibration.

The extracted material parameters were implemented into LS-DYNA 14.0 using *MAT24* and *MAT224* models, with and without the Generalized Incremental Stress-State-Dependent Model (*GISSMO*). A comprehensive simulation study was performed using different element types and formulations, and the results were compared to experimental data. The following conclusions were drawn:

1. The developed failure surface and material parameters allow accurate simulation of guardrail steel fracture under complex loading conditions.
2. *GISSMO* played a critical role in improving prediction accuracy, particularly under multiaxial stress states, and its coupling with *MAT24* and *MAT224* resulted in significantly better agreement with experimental results.
3. While both *MAT224* and *GISSMO* can implement the failure surface, slight differences in response are due to *GISSMO*’s ability to couple damage evolution with flow stress, which *MAT224* lacks.
4. Fully integrated shell elements (*FI*) outperformed under-integrated (*UI*) ones in capturing experimental trends, though shell elements alone were insufficient for capturing complex stress states.
5. Solid elements (*UI* and *FI*) proved more appropriate in multiaxial loading scenarios, with UI solids recommended for their balance of accuracy and computational efficiency.

Importantly, the study provides a validated modeling framework and recommended LS-DYNA 14.0 inputs that can be directly and efficiently implemented in future roadside safety simulations. All inputs were derived from physical testing and constructed without arbitrary fitting, ensuring both transparency and practical utility. Ongoing research will focus on mesh regularization to ensure solution consistency across varying element sizes, a common challenge in FE modeling. In parallel, validation against full-scale crash testing will be pursued to further strengthen the model’s applicability to real-world roadside safety design and evaluation.

## Figures and Tables

**Table 1:** AASHTO M-180 and selected material mechanical properties.

Material	Yield Strength ksi (MPa)	Ultimate Strength ksi (MPa)	Elongation (% in 2 in.)
AASHTO Specification M-180 Minimum Values	50 (345)	70 (483)	12%
Median Values of Compiled Material Available on the Market	63.2 (436)	74.8 (516)	27.1%
Selected Material Mean Values from Material Certificate	65 (448)	74 (510)	30.5%

**Table 2:** Summary of test results, specimens 1–13.

Specimen No.	Yield Strength (MPa)	Ultimate Strength (MPa)	Effective Strain at Failure (DIC)
1	444	547	0.82
2	488	567	0.63
3	499	582	0.76
4	533	615	0.54
5	432	527	1.45
6	489	567	1.22
7	498	595	1.13
8	530	624	1.23
9	546	640	0.82
10	587	678	1.40
11	445	561	1.04
12	503	599	0.98
13	538	633	0.57

**Table 3:** Summary of test results, specimens 14–18, 20, and 21.

Specimen No.	Maximum Force (kN)	Maximum Displacement (mm)
14	250	7.165
15	18.7	6.330
16	18.2	4.536
17	18.2	1.666
18	59.6	NA
20	13.3	5.552
21	13.8	5.248

**Table 4:** Basic input material parameters.

Parameter	Value
Mass density (kg/m^3^)	7850
Elastic modulus (MPa)	1.993 × 10^5^
Yield strength (MPa)	439.6
Poisson’s ratio	0.295
Plastic failure strain	1.400

**Table 5:** Input plasticity curve.

Effective Plastic Strain	Effective Stress (GPa)
0.000	0.4396
0.005	0.4634
0.025	0.5174
0.040	0.5419
0.075	0.5807
0.145	0.6278
0.300	0.6870
0.500	0.7329
0.700	0.7652
0.900	0.7903
1.000	0.8010
1.200	0.8201
1.400	0.8365

## Data Availability

Data is available on request due to restrictions. The data presented in this study are available on request from the corresponding author. The data is not publicly available because this study is part of an ongoing large research project.

## List of Abbreviations

Note: Other variables are unique to specific material models in LS-DYNA 14.0 and are defined where they first appear in the text.

Abbreviation	Definition
AASHTO	American Association of State Highway and Transportation Officials
ASTM	American Society for Testing and Materials
MASH	Manual of Assessing Safety Hardware
FEA	Finite Element Analysis
LS-DYNA	Livermore Software Technology Corporation—Dynamic Nonlinear Analysis (FEA Software)
σ	Cauchy stress tensor
$\sigma_{H}$	Hydrostatic stress
s	Deviatoric stress
$tr\left(\sigma \right)$	Trace of the stress tensor, which is the sum of its diagonal components
***I***	The second-order identity tensor
*P*	Pressure
$I_{1},\;I_{2},\;I_{3}$	Stress invariants
s:s	double contraction of the deviatoric stress tensor with itself
$\sigma_{m}$	Von Mises or average stress
η	Stress triaxiality
ξ	Lode parameter
$J_{3}$	Third deviatoric stress invariant
DIC	Digital Image Correlation
$\sigma_{T}$	True uniaxial stress
$\varepsilon_{T}$	True uniaxial strain
K, $\varepsilon_{o}$, and n	Swift plasticity model constants
MAT224	Tabulated Johnson–Cook LS-DYNA’s material model
MAT24	Piecewise Linear Plasticity LS-DYNA’s material model
GISSMO	Generalized Incremental Stress-State-Dependent Model
ELFORM	Element formulation in LS-DYNA
FI	Fully Integrated elements
UI	Under Integrated elements
